# Supplementary material for: Translocated Legionella pneumophila small RNAs mimic eukaryotic microRNAs targeting the host immune response
Source: Nat Commun. 2022 Feb 9;13:762. doi: 10.1038/s41467-022-28454-x (PMC8828724; doi:10.1038/s41467-022-28454-x)
Supplement: Supplementary file 1 — Supplementary Information [file 41467_2022_28454_MOESM1_ESM.pdf]

Supplementary material

Translocated *Legionella pneumophila* small RNAs mimic eukaryotic microRNAs targeting the host immune response

Tobias Sahr[1], Pedro Escoll[1], Christophe Rusniok[1], Sheryl Bui[2], Gérard Pehau-Arnaudet[3], Gregory Lavieu[2], and Carmen Buchrieser[1*]

[1]Institut Pasteur, Université de Paris, Biologie des Bactéries Intracellulaires and CNRS UMR 3525, 75724, Paris, France, [2] Université de Paris, INSERM ERL U1316, UMR 7057/CNRS, Paris, France and [3]Unité de Technologie et Service BioImagerie Ultrastructurale and CNRS UMR 3528, Paris, France

Supplementary Table 1. Oligonucleotides and probes used in this study

Supplementary Table 2: Human miRNAs known to interact with Ago2 identified via CLIP-seq

Supplementary Figure 1: Evaluation of the SSC resolution to analyse the size-related parameters of DiD-labelled purified *Lp*-MVs.

Supplementary Figure 2: Fluorescent *in situ* hybridization targeting RsmY-RNA molecules in hMDM cells infected with *Lp*-EVs or with *Lp*-bacteria

Supplementary Figure 3: *Lp*-EVs are shed during infection, travel to lysosomal compartments

Supplementary Figure 4: RsmY downregulates proteins implicated in the RIG-I signalling pathway

Supplementary Figure 5: Influence of RsmY and IFN-ß on *L. pneumophila* replication in THP-1 cells depending on RsmY.

Supplementary Figure 6: Extracellular IFN-ß levels after treatment with TLR agonists mimicking bacterial signals are modulated by *Lp*-EVs

Supplementary Table 1. Oligonucleotides and probes used in this study

oligonucleotides used	
T7-RsmY_F	agctgtaatacgactcactatagatacagggatgacatggatatg
RsmY-BglII	agaccacagatctatacagggatgacatggatatg
RsmY-HindIII	tatcgataagcttagaaaggtatactaatgtaaaattagagaataagtgc
T7-as-tPhe_F	tgtaatacgactcactatagagaaaggtatactaatgtaaattagag
as-tPhe -BglII	agaccacagatctagaaaggtatactaatgtaaattagag
as-tPhe -HindIII	tatcgataagcttatacagggatgacatggatatgtc
RsmY1-FISH	auacagggaugacauggauaug
RsmY2-FISH	agggaccaguacaaaggauguac
T7-tPhe_F	agctgtaatacgactcactatagggccagatagctcagtcg
tPhe-BglII	agaccacagatctggccagatagctcagtcg
tPhe-HindIII	tatcgataagcttgattctaatggtggccagagg
T7-as-tPhe_F	tgtaatacgactcactatagggtaactcattgattctaatggtg
as-tPhe -BglII	agaccacagatctaatggtggccagaggcagaatcg
as-tPhe -HindIII	tatcgataagcttggccagatagctcagtcgg
T7-ctrlRNA_F	agctgtaatacgactcactatagatacaatgcaagatgaagaagagc
ctrlRNA-BglII	agaccacagatctgatacaatgcaagatgaagaagagc
ctrlRNA-HindIII	tatcgataagcttagcttcatcatcagtaaactctgg
T7-DDx58_F	agctgtaatacgactcactatagatgaatgaatggaatgttggg
DDx58_R	ccatttgtattgggtcttagtaag
T7-Rel_F	agctgtaatacgactcactatagtataacttgcaagatttaaatcc
Rel_R	tccagggaagagatgatggtgg
T7-Irak1_F	agctgtaatacgactcactatagatgtgttcacctgggcagatc
Irak1-R	actcaaggacaacctggacgtc
Mir144-BglII	agaccacagatcttggggccctggctggg
Mir144-HindIII	tatcgataagcttgggggtgcccggactag
Mir146-BglII	agaccacagatctccgatgtgtatcctcagctttg
Mir146-HindIII	tatcgataagcttacgatgacagagatatcccagc
H1-sRNA-BamHI_F	ctctagaactagtggatcccc
H1-sRNA-BamHI_R	actcctcggatcctcgaggtcgacggtatcg
DDx58-MssI	attctagttgtttaaacgatgaatgaatggaatgttggg
DDx58-XhoI	ctctagactcgagccatttgtattgggtcttagtaag
Rel-MssI	attctagttgtttaaacgtataacttgcaagatttaaatcc
Rel-XhoI	ctctagactcgagtccagggaagagatgatggtgg
Irak1-MssI	attctagttctttaaacgatgtgttcacctgggcagatc
Irak1-XhoI	ctcagactcgagactcaaggacaacctggacgtc

Supplementary Table 2 Human miRNAs known to interact with Ago2 identified via CLIP-seq

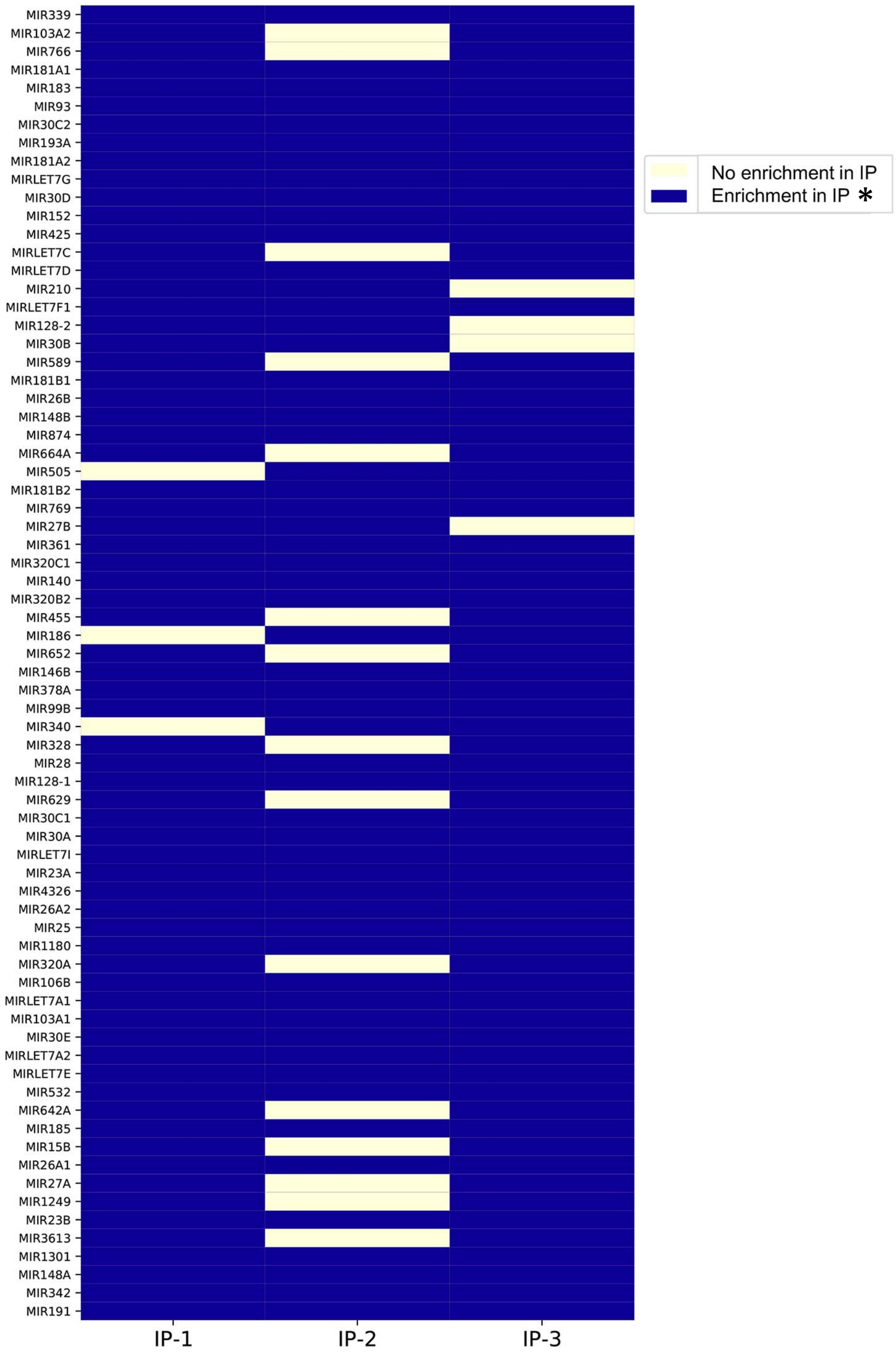

* A mi-RNA was counted as enriched and present in the Ago2 CLIPseq if it was enriched in at least two of the three experiments.

Supplementary Figure 1

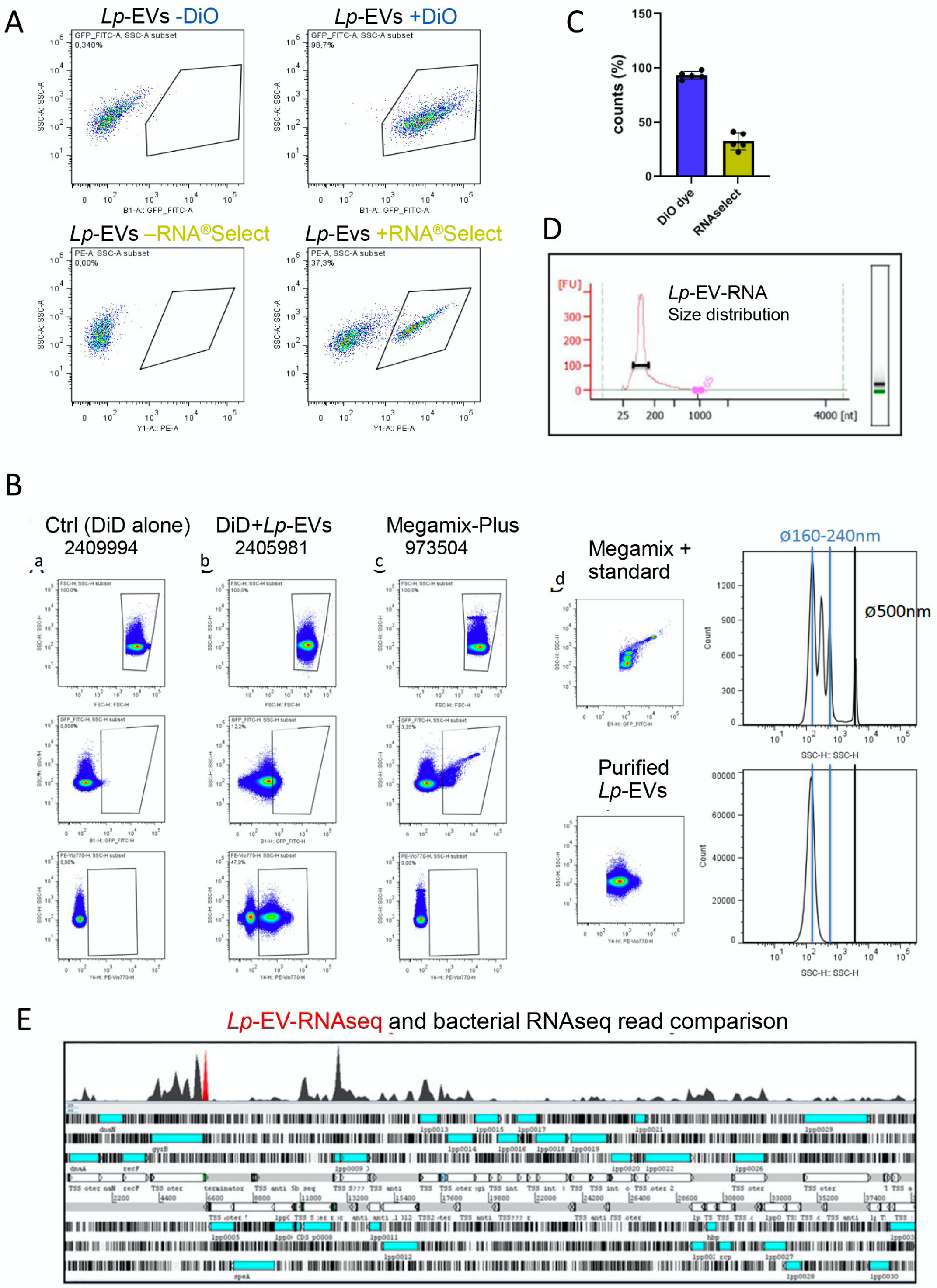

Supplementary Figure 1. RNA content of *Lp*-EVs and evaluation of the SSC resolution to analyse the size-related parameters of DiD-labelled purified *Lp*-EVs. A) Purified *Lp*-EVs were independently labelled either with DiO dye (lipids) or with Syto-RNAselect (RNA) and then analyzed by flow cytometry. *Events* from the forward scatter (FSC) versus side scatter (SSC) plot were included in the fluorescence analysis gate shown. Positive fluorescent signals were distinguished by comparing with non-labelled control *Lp*-EVs. **B)** Representative flow cytometry results showing labelling and gating strategies to evaluate the SSC resolution of purified *Lp*-EVs to analyse size-related parameters. **a)** Column A shows a representative control experiment using DiD dye in PBS alone, to be able to

distinguish *Lp*-EVs from the background noise. **b)** Colum B shows *Lp*-EVs labelled with DiD dye (PE-Vio770-H channel. **c)** Optimization of the cytometer settings for discriminating the 4 individual peaks of the green, fluorescent Megamix-Plus SSC beads (GFP-FITC-H channel). **d)** Adjustments were used to characterize stained standardization beads and *Lp*-EVs, to determine the approximate diameter represented as a side scatter SSC-H x count histogram. **C)** Flow cytometry results as (mean) SD of 5 independent biological replicates. **D)** Representative image (Bioanalyser) obtained for the RNA isolated from purified *Lp*-EVs showing their size distribution. **E)** Representation of a section of the genome of *L. pneumophila* Paris (Artemis analyses tool) showing the read coverage of the *Lp*-EVseq data (red) and the bacterial RNAseq control results (black). The comparison highlights the accumulation of the RsmY-RNA transcript in the *Lp*-EV fraction (red peak) compared to the bacterial fraction (black peaks). Source data provided as Source data file.

Supplementary Figure 2

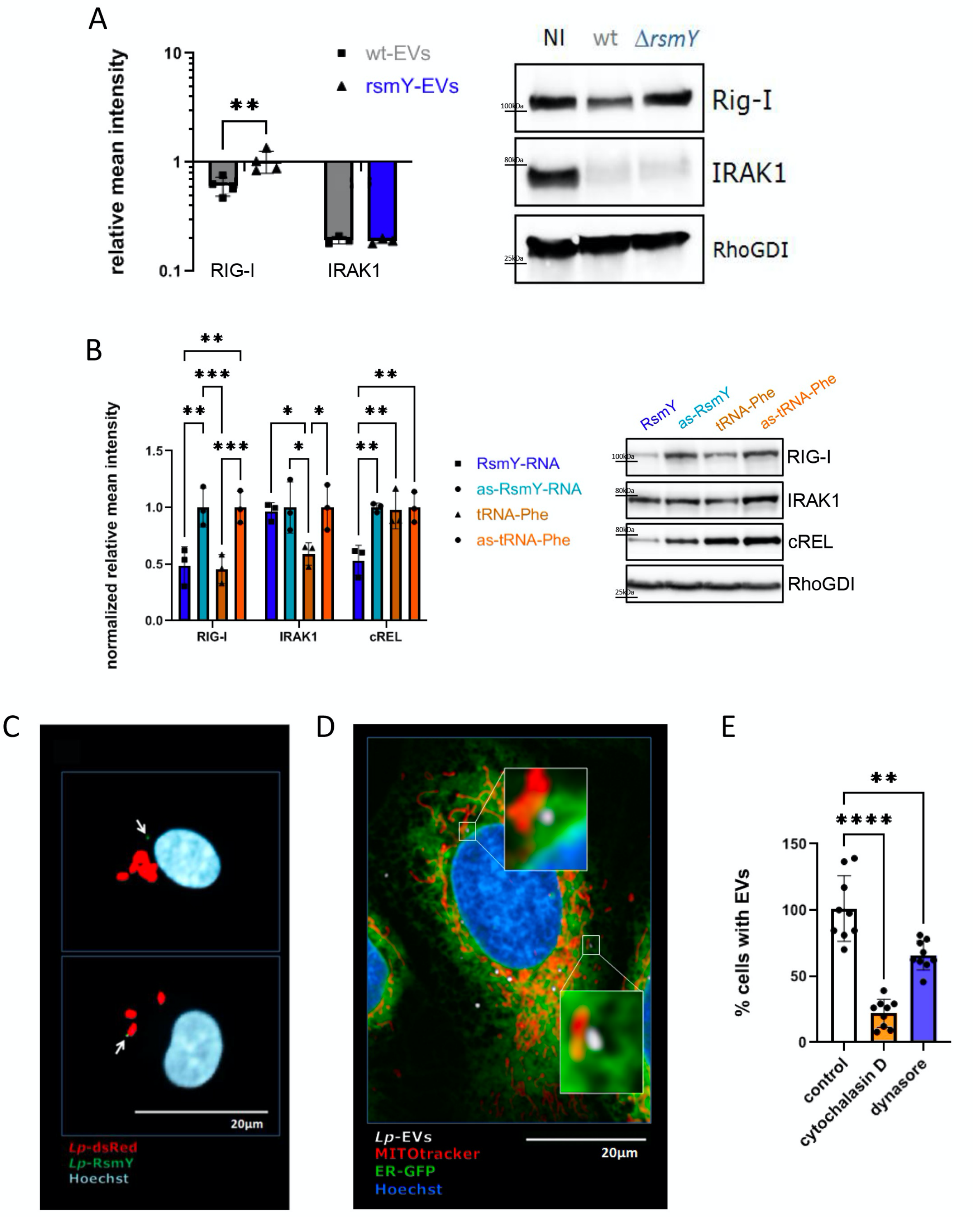

Supplementary Figure 2. Fluorescent *in situ* hybridization targeting RsmY-RNA molecules in hMDM cells incubated with *Lp*-EVs or infected with *Lp*-bacteria. **(A)** hMDM cells incubated for 3h with wt or *ΔrsmY Lp*-EVs. Quantification of RIG-I and IRAK protein levels relative to non-infected (NI) and RhoGDI loading control. Data presented as (mean) SD values of n≥3 independent biological replicates using a two-way ANOVA analysis with ** p=0.0073. Right, representative western blot. **B)** Quantification of RIG-I, IRAK1 and cRel protein levels after THP-1 transfection with RsmY or tRNA-Phe RNAs. Control, *in vitro* transcribed anti-sense strands, as-RsmY, as-tRNA-Phe. Relative mean intensities normalized to control values and RhoGDI loading control. Representative

blots of RIG-I, IRAK1 and cRel levels of cells transfected with RsmY or tRNA-Phe or their anti-sense sequences. Data are presented as (mean) SD of n=3 independent biological replicates using a two-way ANOVA analysis with values of $p<0.05$ significant (*), $p<0.01$ very significant (**) and $p<0.001$ extremely significant (***). **C**) Representative image using FISH probes specific for RsmY (green) visualizing *Lp*-EVs shed from bacteria (red) (3 independent experiments). Blue, nucleus fluorescently stained (Hoechst 33342) **D)** U2OS cells stably expressing Sec61β-GFP (ER green, mitochondria red, nucleus blue) were incubated with purified *Lp*-EVs stained with DiD dye (white). Representative image of at least 3 independent experiments. **E)** Entry of *Lp*-EVs into U2OS-Sec61β cells. Purified *Lp*-EVs were labelled with DiD dye and the internalization into U2OS-Sec61β cells was followed by live cell image acquisition of multiple fields per well over time at 37°C (5% CO_2) on an automated confocal microscope. U2OS-Sec61β cells containing *Lp*-EVs were counted in absence or presence of 1µM cytochalasin D or 80µM dynasore. Relative percentage of cells containing *Lp*-EVs, bars represent the (mean)SD of n=9 independent biological experiments. For statistical analysis, a two-way ANOVA was performed with values of $p<0.01$ very significant (**) and and $p<0.0001$ (****). (Exact p values: $p<0.0001$ and $p=0.0010$). Source data provided as Source data file.

Supplementary Figure 3

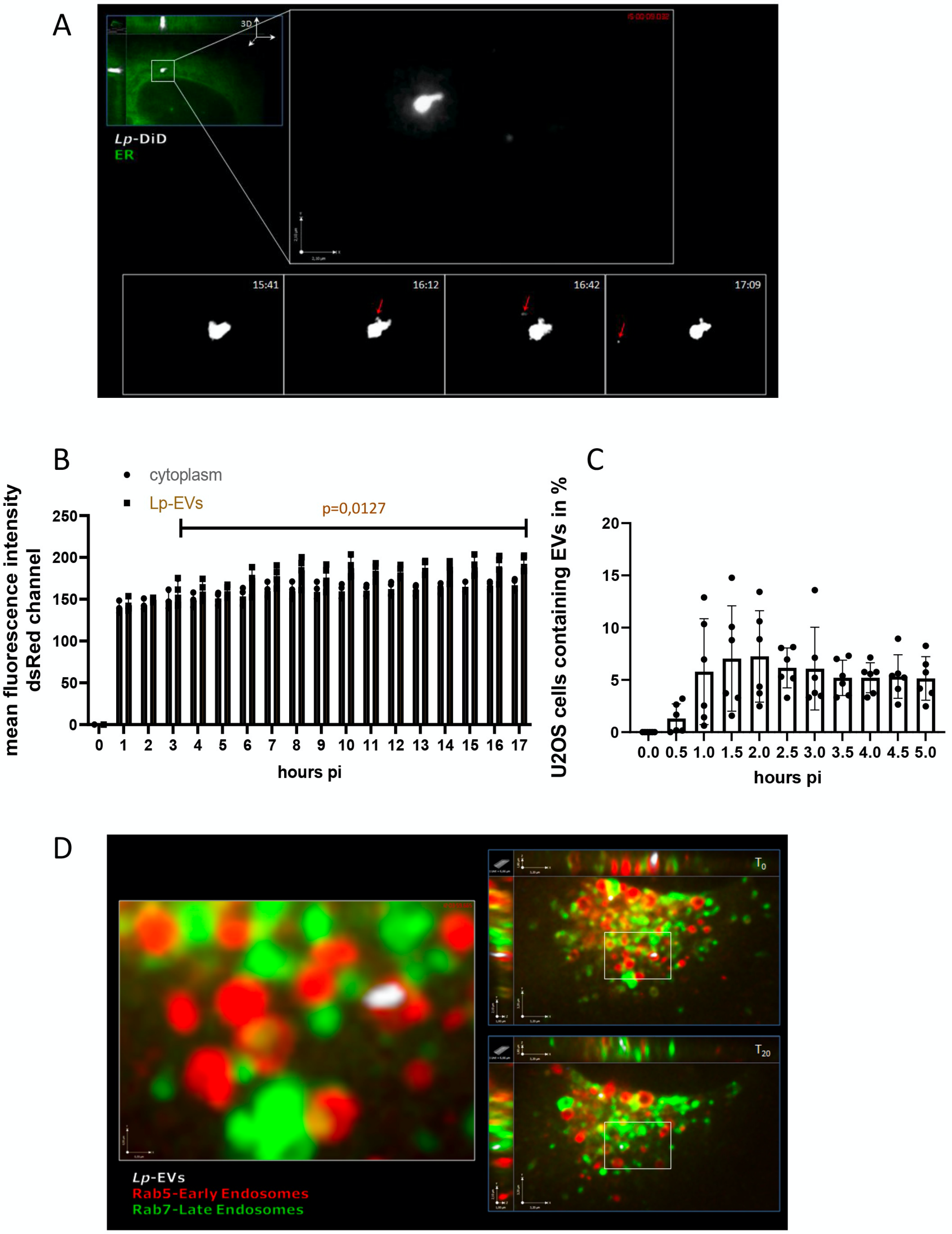

Supplementary Figure 3: ***Lp*****-EVs are shed during infection and travel to lysosomal compartments. A)** Freeze images taken from the 3D Confocal Time-Lapse Movie 1 of a U2OS Sec61β-GFP cells infected with DiD-labelled *L. pneumophila* (white). Images below highlight by a red arrow a potential EV shed by *L. pneumophila* after infection of an U2OS-Sec61β-GFP cell. **B)** Quantification of co-localization events over time. Live cell image acquisition of multiple fields per well of infected hMDM cells was performed over time at 37°C (5% CO2) on an automated confocal microscope and the mean fluorescence intensity (MFI) of pHrodo in detected *Lp*-EVs was analysed by

HCA, showing a significant enrichment of MFI in *Lp*-EVs*,* suggesting that they co-localize with acidic organelles also in hMDM cells. Data are presented as (mean) SD with each dot represents the mean of around 200 independently analysed cells. For statistical analysis, an unpaired t-test was performed with two-tailed values of $p<0.05$ significant. Source data provided as Source data file. **C)** U2OS cells were infected with DiD-labelled *Lp*-EVs purified from *L. pneumophila* wt broth cultures grown until post-exponential phase (OD4.2). Live cell image acquisition of multiple fields per well was performed over time at 37°C (5% CO_2) on an automated confocal microscope to analyse percentage of cells that had taken up *Lp*-EVs over time. Data are presented (mean) SD with each dot represents the mean of up to 650 independently analysed cells. Source data provided as Source data file. **D**) Freeze images taken from the 3D Confocal Time-Lapse Movie 2 of a U2OS-Sec61β-GFP cell infected with DiD-labelled *Lp*-EVs (white). *Lp*-EVs co-localize with Rab5a (red) taken as marker for early endosomes at T0. Over time (T20) the same *Lp*-EV colocalizes with Rab7A (green), taken as marker for late endosomes.

Supplementary Figure 4

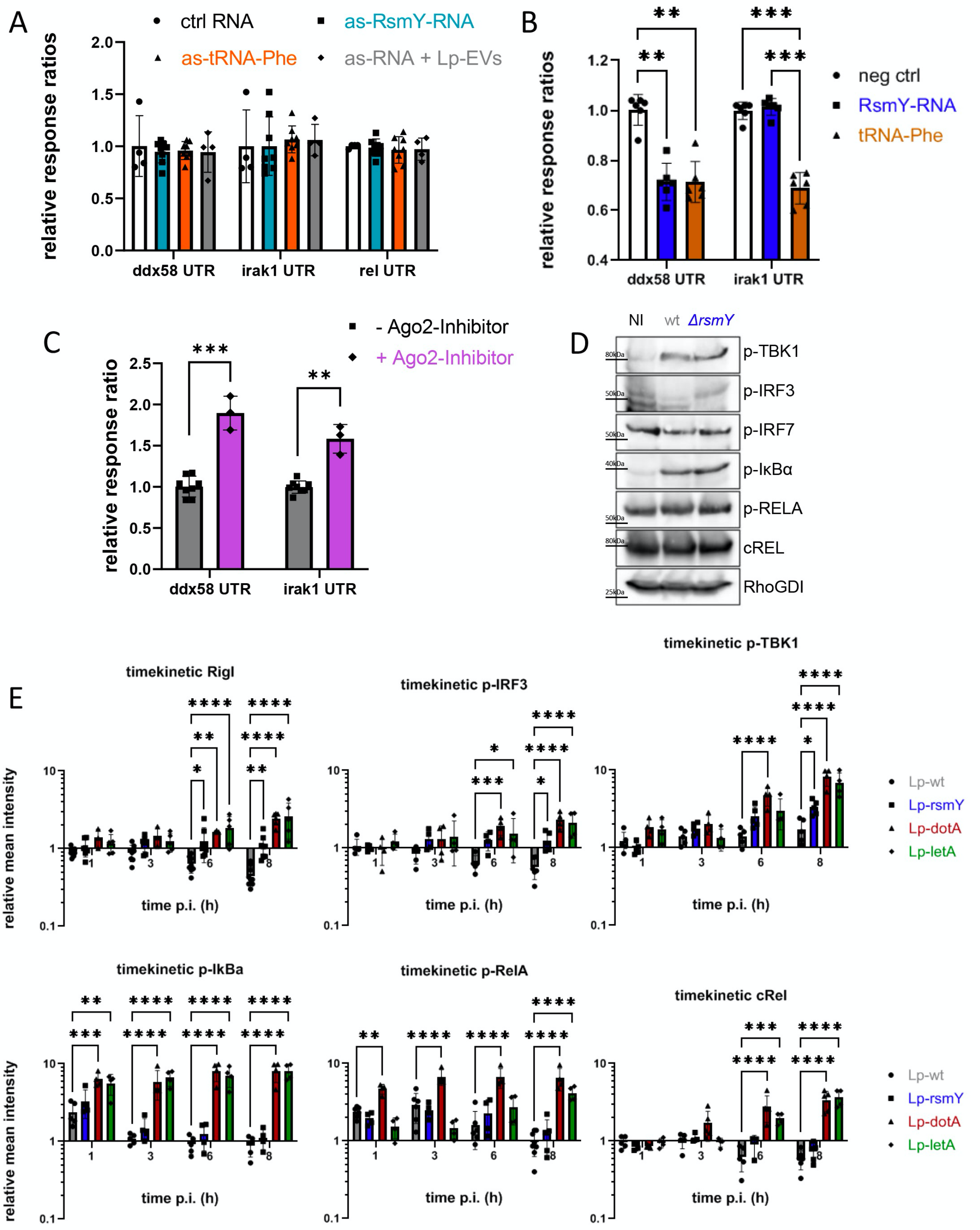

Supplementary Figure 4: RsmY downregulates proteins implicated in the RIG-I signalling pathway. A) Dual luciferase reporter assay control experiments, using anti-sense of RsmY and tRNA-Phe-RNA. Results are shown as relative luciferase activity of transfected THP1 cells; the ctrl RNA is defined as 100 %. The bars represent the (mean) SD of n≥3 independent biological repeats. Source data provided as Source data file. **B**) Luciferase activities of transfected CD14+ in presence of

the ctrlRNA, or RsmY or tRNA-Phe RNAs, respectively. Results are shown as relative luciferase activity (where luciferase activity in CD14+ cells transfected with ctrl RNA is defined as 100 %. . Bars show the (mean) SDof n=6 independent experiments. For statistical analysis, a two-way ANOVA with values of p<0.05 significant (*), p<0.01 very significant (**) and p<0.001 extremely significant (***) was performed. Source data provided as Source data file. **C)** Relative luciferase activities of THP-1 cells treated or not with Ago2 inhibitor in presence of the UTR of ddx58 (RIG-I) and irak1.The bars represent the (mean) SD of at least 3 independent biological repeats. For statistical analysis, a two-way ANOVA with values of p<0.05 significant (*), p<0.01 very significant (**) and p<0.001 extremely significant (***) was performed. Source data provided as Source data file. **D)** Representative western blot used in the quantification of Figure 4C. The blots show the protein levels of cRel and RhoGDI (loading control) and the phosphorylation status of proteins TBK-1, IRF-3, IRF-7, IkBa and RelA in non-infected THP-1 cells and THP-1 cells infected for 3h with *Lp*-EVs originating from wt or the Δ*rsmY*-mutant**. E)** Quantitative analyses of the protein levels of key proteins of the RLR and TLR signalling pathway analysed after 1h, 3h, 6h and 8h post-infection with wt (grey), Δ*rsmY* (blue), Δ*letA* (green) or Δ*dotA* (red) infected THP-1 cells. The bars represent the (mean) SD of at least 3 independent biological repeats normalized against the value of non-infected cells and the RhoGDI loading control using two-way ANOVA analysis with values of p<0.05 significant (*), p<0.01 very significant (**) and p<0.001 extremely significant (***) and p<0.0001 (****). Source data provided as Source data file.

Supplementary Figure 5

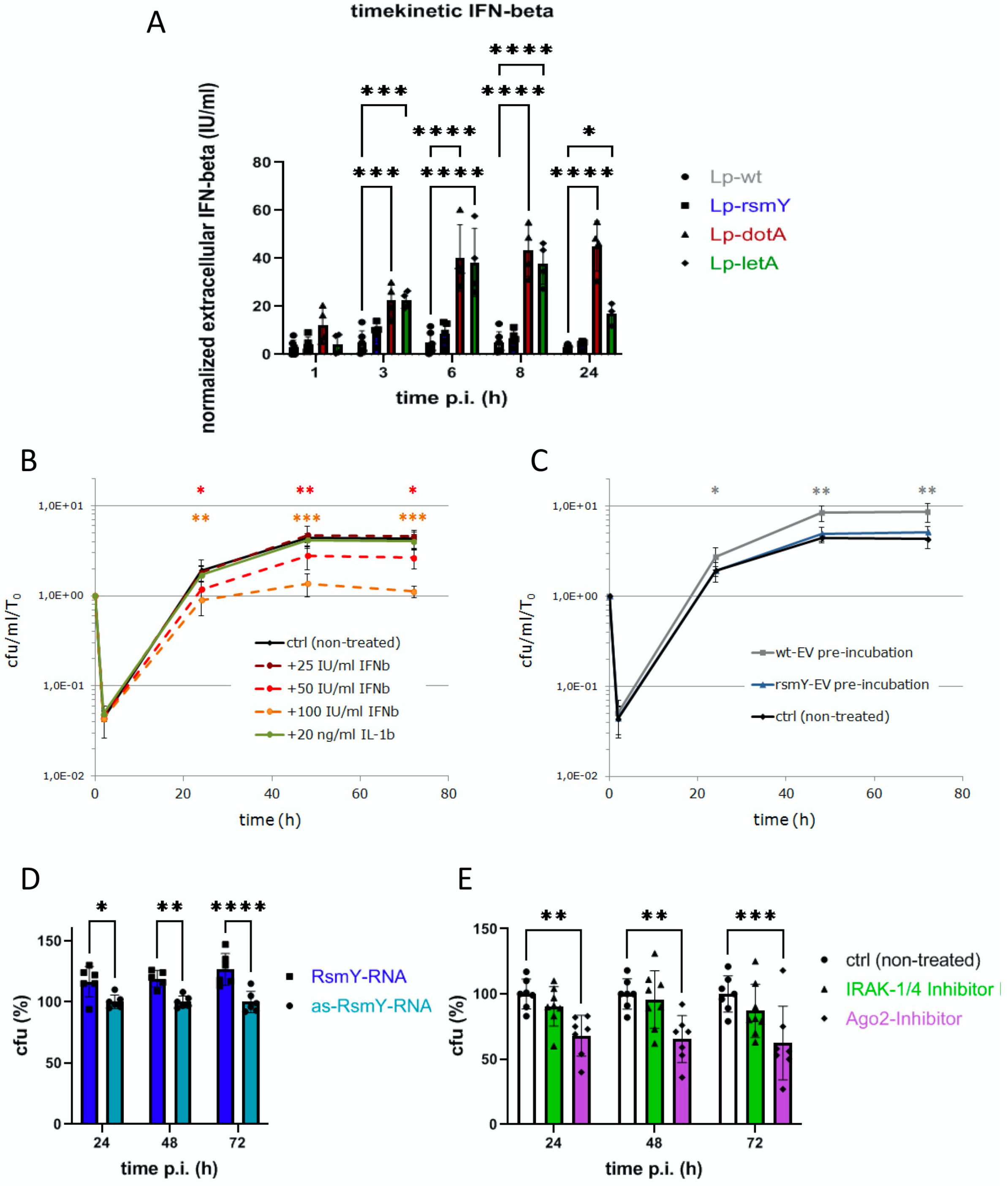

Supplementary Figure 5. RsmY and IFN-ß concentrations influence *L. pneumophila* replication in THP-1 cells. **A)** *L. pneumophila* wt, *ΔrsmY*, *ΔletA* and *ΔdotA* strains were grown until post-exponential phase (OD4.2) and THP-1 cells were infected for 1h, 3h, 6h, 8h and 24h. Extracellular IFN-ß concentrations were quantified and normalized against non-infected control cells. Data are presented as (mean) SD of at least n=4 biological repeats using two-way ANOVA analysis of p<0.05 significant (*), p<0.01 very significant (**) and p<0.001 extremely significant (***) and p<0.0001 (****). Source data are provided with this paper. **B, C**) Intracellular growth of *L. pneumophila* wt **B**) at increasing concentration of extracellular IFN-ß (0, black; 25/IU/ml dark red; 50 IU/ml red; 100IU/ml, orange). or at 20ng/ml Interleukin-1ß (green) added to the infection medium. **C)** Pre-

incubation of THP-1 cells with purified wt-*Lp*-EVs (grey), Δ*rsmY*-EVs (blue) or not EV-treated (black) conditions. Bars show the (mean) SD of colony forming units (cfu) per ml normalized to timepoint T0 of at least 4 independent experiments using an unpaired t-test for statistical analysis with two-tailed values of $p<0.05$ significant, $p<0.01$ very significant (**) and $p<0.001$ extremely significant (***). ***D***) THP-1 cells were transfected with *in vitro* transcribed RsmY-RNA or the antisense RNA of RsmY as control and infected with *Legionella pneumophila* wt bacteria. The graph shows the percentage of colony forming units (% cfu) at 24, 48 and 72h post-infection relative to the as-RsmY control. The bars indicate the (mean) SD of at least 5 independent experiments using two-way ANOVA analysis with $p<0.05$ significant (*), $p<0.01$ very significant (**) and $p<0.0001$ (****). Source data provided as Source data file. **E**) THP-1 cells were pre-incubated with inhibitors of IRAK1/4 (green), Ago2 (purple) or not (white) before infection with *L. pneumophila* wt bacteria. The graph shows the percentage of the colony forming units (% cfu) of *Lp*-wt after 24, 48 and 72h post-infection relative to the non-treated control. The bars indicate the (mean) SD of at least 7 independent experiments using a two-way ANOVA analysis with $p<0.05$ significant; $p<0.01$ (**) and $p<0.001$ (***). Source data provided as Source data file.

Supplementary Figure 6

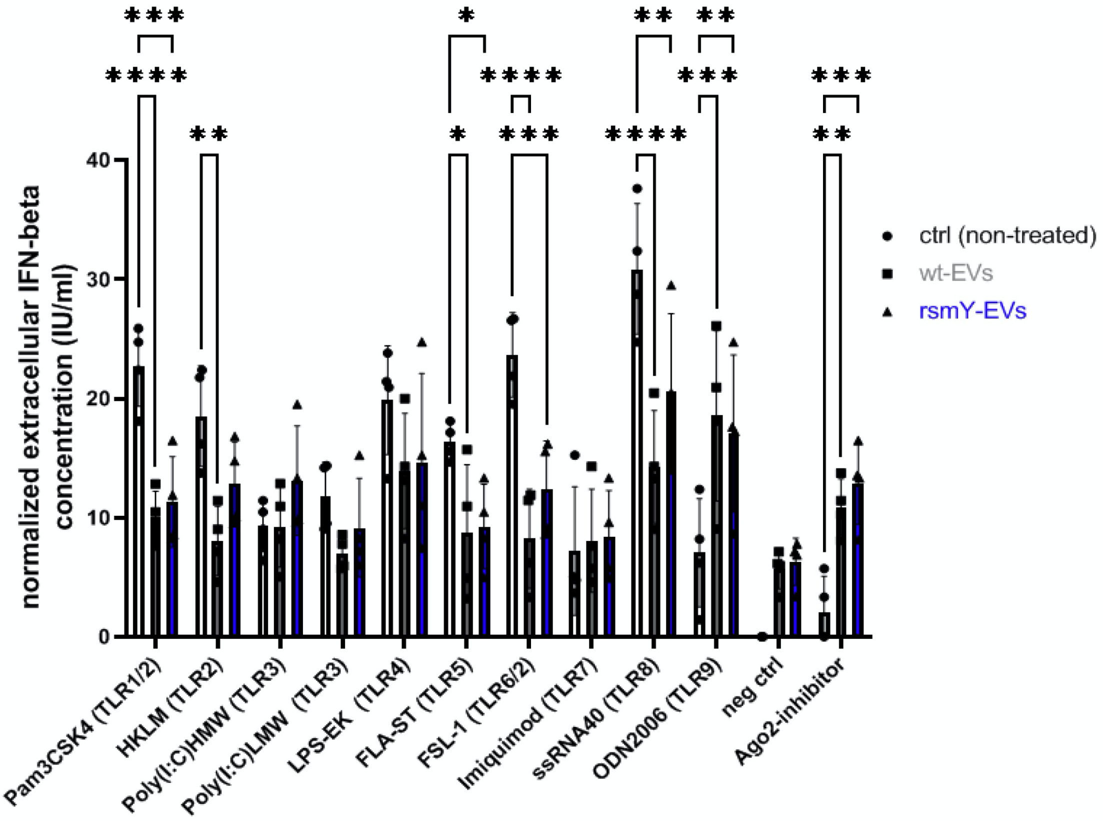

Supplementary Figure 6. **Extracellular IFN-ß levels after treatment with TLR agonists mimicking bacterial signals are modulated by *Lp*-EVs**. THP-1 cells were pre-treated for 3h with or without *Lp*-EVs purified from *L. pneumophila* wt or from the Δ*rsmY* strain. Subsequently, TLR-related agonists were added and the extracellular IFN-ß concentrations were measured by ELISA 20h post incubation showing that particularly TLR signaling pathways depending on IRAK1 like TLR1, TRL2, TLR6 or on RLR (Poly(I:C), ssRNA40) Additionally, extracellular concentrations of IFN-b of *Lp*-EV-treated THP-1 cells after Ago2-Inhibition was analyzed. The graphs show the mean of n=4 biological repeats with bars represent the (mean) SD of the extracellular IFN-b concentration relative to the non-pre-treated negative control. For statistics, a two-way ANOVA analysis was performed with values of $p<0.05$ significant (*), $p<0.01$ very significant (**) and $p<0.001$ extremely statistically significant (***) and $p<0.0001$ (****). Source data provided as Source data file.
