# Supplementary material for: Translocated Legionella pneumophila small RNAs mimic eukaryotic microRNAs targeting the host immune response
Source: Nat Commun. 2022 Feb 9;13:762. doi: 10.1038/s41467-022-28454-x (PMC8828724; doi:10.1038/s41467-022-28454-x)
Supplement: Supplementary file 3 — Description of Additional Supplementary Files [file 41467_2022_28454_MOESM3_ESM.pdf]

Description of Additional Supplementary Files

Supplementary Data 1: Thirty-nine RNA molecules identified as enriched in Lp-EVs.

Supplementary Movie 1: related to Supplementary Figure 3A

3D Confocal Time-Lapse Movie of a U2OS Sec61β-GFP cell infected with *L. pneumophila* labelled with DiD dye. The movie shows during a time course of 18 min (one image every 30 sec) a DiD-stained *L. pneumophila* bacterium (in white) that sheds several potential *Lp*-EVs during infection.

Supplementary Movie 2: related to Supplementary Figure 3B

3D Confocal Time-Lapse Movie of a U2OS *cell labelled with Rab5 and Rab7 CellLight and incubated with DiD-labelled Lp-EVs purified from L. pneumophila wt grown until post-exponential phase (OD=4.2). The video shows during 20 min (one image every minute) a single Lp-EV contained within a red Rab5 early endosome that is progressively maturated to a green Rab7 late endosome.*
