# Supplementary material for: Translocated Legionella pneumophila small RNAs mimic eukaryotic microRNAs targeting the host immune response
Source: Nat Commun. 2022 Feb 9;13:762. doi: 10.1038/s41467-022-28454-x (PMC8828724; doi:10.1038/s41467-022-28454-x)
Supplement: Supplementary file 7 — Reporting Summary [file 41467_2022_28454_MOESM7_ESM.pdf]

nature portfolio

Corresponding author(s): Carmen Buchrieser

Last updated by author(s): 2021/12/08

Reporting Summary

Nature Portfolio wishes to improve the reproducibility of the work that we publish. This form provides structure for consistency and transparency in reporting. For further information on Nature Portfolio policies, see our Editorial Policies and the Editorial Policy Checklist.

Statistics

For all statistical analyses, confirm that the following items are present in the figure legend, table legend, main text, or Methods section.

n/a	Confirmed	
☐	☒	The exact sample size (n) for each experimental group/condition, given as a discrete number and unit of measurement
☐	☒	A statement on whether measurements were taken from distinct samples or whether the same sample was measured repeatedly
☐	☒	The statistical test(s) used AND whether they are one- or two-sided<br>*Only common tests should be described solely by name; describe more complex techniques in the Methods section.*
☐	☒	A description of all covariates tested
☐	☒	A description of any assumptions or corrections, such as tests of normality and adjustment for multiple comparisons
☐	☒	A full description of the statistical parameters including central tendency (e.g. means) or other basic estimates (e.g. regression coefficient) AND variation (e.g. standard deviation) or associated estimates of uncertainty (e.g. confidence intervals)
☐	☒	For null hypothesis testing, the test statistic (e.g. F, t, r) with confidence intervals, effect sizes, degrees of freedom and P value noted<br>*Give P values as exact values whenever suitable.*
☒	☐	For Bayesian analysis, information on the choice of priors and Markov chain Monte Carlo settings
☒	☐	For hierarchical and complex designs, identification of the appropriate level for tests and full reporting of outcomes
☒	☐	Estimates of effect sizes (e.g. Cohen's d, Pearson's r), indicating how they were calculated

Our web collection on statistics for biologists contains articles on many of the points above.

Software and code

Policy information about availability of computer code

Data collection	no software was used for data collections
Data analysis	Data analysis was performed using GraphPad Prism program, version 9.3.0; Sequence analyses was udnertaken with Cutadapt software version 1.15; https://github.com/najoshi/sickle 1.33; Bowtie 2.3.4.3; Samtools suite 1.3 https://github.com/samtools/samtools; Sartools version 1.3.0 ; Artemis analysing tool 16.0.17; Velocity 6.5.1; Columbus 2.9.1

For manuscripts utilizing custom algorithms or software that are central to the research but not yet described in published literature, software must be made available to editors and reviewers. We strongly encourage code deposition in a community repository (e.g. GitHub). See the Nature Portfolio guidelines for submitting code & software for further information.

Data

Policy information about availability of data

All manuscripts must include a data availability statement. This statement should provide the following information, where applicable:

- Accession codes, unique identifiers, or web links for publicly available datasets
- A description of any restrictions on data availability
- For clinical datasets or third party data, please ensure that the statement adheres to our policy

RNAseq data have been deposited at NCBI Gene Expression Omnibus (GEO) database under accession number GSE159109 (https://www.ncbi.nlm.nih.gov/geo/query/acc.cgi?acc=GSE159109) and GSE190376 (https://www.ncbi.nlm.nih.gov/geo/query/acc.cgi?acc=GSE190376), Flow cytometry data have been deposited at https://flowrepository.org/ under accession numbers FR-FCM-Z2XL and FR-FCM-Z2XM, human genome sequence build GRCh38, http://www.mirbase.org/search.shtml (Release 22.1: October 2018)
http://diana.imis.athena-innovation.gr/DianaTools/index.php?r=microT_CDS/index (Version 5.0) were used

Field-specific reporting

Please select the one below that is the best fit for your research. If you are not sure, read the appropriate sections before making your selection.

☒ Life sciences ☐ Behavioural & social sciences ☐ Ecological, evolutionary & environmental sciences

For a reference copy of the document with all sections, see nature.com/documents/nr-reporting-summary-flat.pdf

Life sciences study design

All studies must disclose on these points even when the disclosure is negative.

Sample size	Not applicable
Data exclusions	No data have been excluded
Replication	Experiments were replicated as stated in the methods section and figure legends but at least 3 times
Randomization	There was no randomization
Blinding	No blinding was performed.

Reporting for specific materials, systems and methods

We require information from authors about some types of materials, experimental systems and methods used in many studies. Here, indicate whether each material, system or method listed is relevant to your study. If you are not sure if a list item applies to your research, read the appropriate section before selecting a response.

Materials & experimental systems

n/a	Involved in the study	
☐	☒	Antibodies
☐	☒	Eukaryotic cell lines
☒	☐	Palaeontology and archaeology
☒	☐	Animals and other organisms
☒	☐	Human research participants
☒	☐	Clinical data
☒	☐	Dual use research of concern

Methods

n/a	Involved in the study	
☒	☐	ChIP-seq
☐	☒	Flow cytometry
☒	☐	MRI-based neuroimaging

Antibodies

Antibodies used

Argonaute 2 (C34C6) Cell Signaling 2897 1:50 https://media.cellsignal.com/coa/2897/2897-coa.pdf
Normal Rabbit IgG Cell Signaling 2792 1:50 https://media.cellsignal.com/coa/2792/2792-coa.pdf
RIG-I (D14G6) Cell Signaling 3743 1:1000 https://media.cellsignal.com/coa/3743/3743-coa.pdf
IRAK1 (D51G7) Cell Signaling 4504 1:1000 https://media.cellsignal.com/coa/4504/4504-coa.pdf
c-Rel Cell Signaling 4727 1:1000 https://media.cellsignal.com/coa/4727/4727-coa.pdf
P-TBK1/NAK (Ser172) (D52C2) Cell Signaling 5483 1:1000 https://media.cellsignal.com/coa/5483/5483-coa.pdf
P-IRF3 (Ser396) (4D4G) Cell Signaling 4947 1:1000 https://www.cellsignal.com/products/primary-antibodies/phospho-irf-3-ser396-4d4g-rabbit-mab/4947
P-IRF7 (Ser471/472) Cell Signaling 5184 1:1000 https://www.cellsignal.com/products/primary-antibodies/phospho-irf-7-ser471-472-antibody/5184
P-IkBa (Ser32/36) (5A5) Cell Signaling 9246 1:1000 https://media.cellsignal.com/coa/9246/9246-coa.pdf
P-NF-kB p65 (Ser536) (93H1) Cell Signaling 3033 1:1000 https://media.cellsignal.com/coa/3033/3033-coa.pdf
Anti-rabbit IgG, HRP-linked Cell Signaling 7074 1:5000 https://media.cellsignal.com/coa/7074/7074-coa.pdf
Anti-mouse IgG, HRP-linked Cell Signaling 7076 1:5000 https://media.cellsignal.com/coa/7076/7076-coa.pdf
RhoGDIa (A20) Santa Cruz Biotechnology sc-360 1:2000 https://www.citeab.com/antibodies/826384-sc-360-rho-gdi-antibody-a-20
Biotin Monoclonal Antibody (Z021) Invitrogen 033700 1:2000 https://www.thermofisher.com/antibody/product/Biotin-Antibody-clone-Z021-Monoclonal/03-3700

Validation

We did not validate any of our antibodies but we relied on the validtaion done by the manufacturer. Please find here each technical data sheet of the manufacturer with the description of the validation
https://media.cellsignal.com/coa/2897/2897-coa.pdf
https://media.cellsignal.com/coa/2792/2792-coa.pdf
https://media.cellsignal.com/coa/3743/3743-coa.pdf
https://media.cellsignal.com/coa/4504/4504-coa.pdf
https://media.cellsignal.com/coa/4727/4727-coa.pdf

https://media.cellsignal.com/coa/5483/5483-coa.pdf
https://www.cellsignal.com/products/primary-antibodies/phospho-irf-3-ser396-4d4g-rabbit-mab/4947
https://www.cellsignal.com/products/primary-antibodies/phospho-irf-7-ser471-472-antibody/5184
https://media.cellsignal.com/coa/9246/9246-coa.pdf
https://media.cellsignal.com/coa/3033/3033-coa.pdf
https://media.cellsignal.com/coa/7074/7074-coa.pdf
https://media.cellsignal.com/coa/7076/7076-coa.pdf
https://www.citeab.com/antibodies/826384-sc-360-rho-gdi-antibody-a-20
https://www.thermofisher.com/antibody/product/Biotin-Antibody-clone-Z021-Monoclonal/03-3700

Eukaryotic cell lines

Policy information about cell lines

Cell line source(s)	Cell lines used were THP-1 purchased from ATCC and U2OS-Sec61β cells cells published in Proc Natl Acad Sci U S A. 2016 Feb 16;113(7):1901-6. doi: 10.1073/pnas.1522067113
Authentication	Standard cell lines were used, no particular authentification process was conducted
Mycoplasma contamination	All cell lines were tested for Mycoplasma contamination and tested negative
Commonly misidentified lines (See ICLAC register)	We did not use any commonly misidentifed cell lines in this study

Flow Cytometry

Plots

Confirm that:

☒ The axis labels state the marker and fluorochrome used (e.g. CD4-FITC).

☒ The axis scales are clearly visible. Include numbers along axes only for bottom left plot of group (a 'group' is an analysis of identical markers).

☒ All plots are contour plots with outliers or pseudocolor plots.

☒ A numerical value for number of cells or percentage (with statistics) is provided.

Methodology

Sample preparation	The samples were prepared as described in the M&M section. L. pneumophila strains were grown in BYE until post-exponential phase (OD4.2). Bacterial cells were harvested by centrifugation at 5.000xg, 4°C for 15 min and the pellet was frozen at -80°C for further use. The supernatant was twice passed through a 0.2µm PES membrane Stericup® Quick Release (Millipore) and re-centrifuged for 15min at 15.000xg, 4°C. The supernatant was treated with RNaseA/T1 (Thermo Scientific) at a final concentration of 2µg/ml RNaseA for 1h at 37°C followed by centrifugation at 150.000xg for 2h at 4°C to pellet the Lp-EVs. The Lp-EV-pellet was washed, re-centrifuged and resuspended in PBS. For incubation experiments with U2OS or hMDM for FISH, the purified Lp-EVs were labelled with Vybrant DiD-dye (Thermo Scientific), 30min at 37°C and subsequently cleaned-up using Exosome Spin Columns (MW3000, Thermo Scientific) for removal of unincorporated dye. Human cells were incubated with Lp-EVs at an MOI of 10 (according to flow cytometry dye-labelled events).
Instrument	MACSQuant flow cytometer and ZetaView® QUATT
Software	The data were analysed with FlowJo software
Cell population abundance	We did not sort any populations thus no post sort fractions details can be given
Gating strategy	Figure S1B shows representative flow cytometry results showing labelling and gating strategies to evaluate the SSC resolution of purified Lp-EVs to analyse size-related parameters. a) Column A shows a representative control experiment using DiD dye in PBS alone, to be able to distinguish Lp-EVs from the background noise. b) Colum B shows Lp-EVs labelled with DiD dye (PE-Vio770-H channel. c) Optimization of the cytometer settings for discriminating the 4 individual peaks of the green fluorescent Megamix-Plus SSC beads (GFP-FITC-H channel). d) Adjustments were used to characterize stained standardization beads and Lp-EVs, to determine the approximate diameter represented as a side scatter SSC-H x count histogram

☒ Tick this box to confirm that a figure exemplifying the gating strategy is provided in the Supplementary Information.
