# Supplementary material for: Translocated Legionella pneumophila small RNAs mimic eukaryotic microRNAs targeting the host immune response
Source: Nat Commun. 2022 Feb 9;13:762. doi: 10.1038/s41467-022-28454-x (PMC8828724; doi:10.1038/s41467-022-28454-x)

Bacterial infection, RIG-I, Fig1G

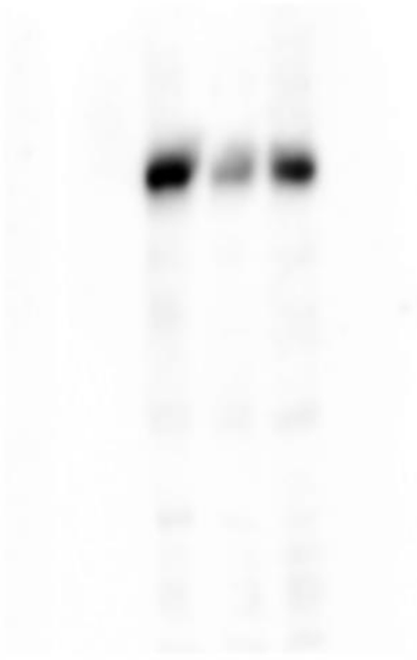

Bacterial infection, IRAK1 (left), Fig1G

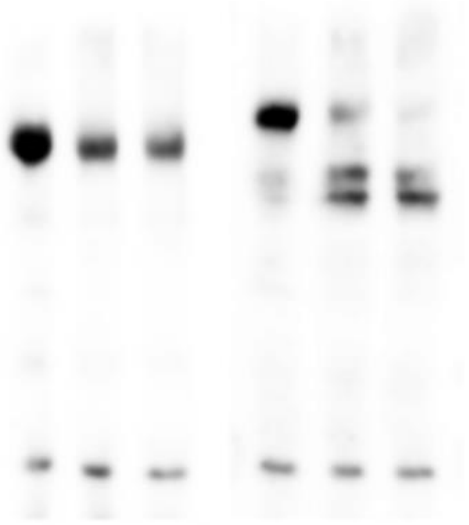

Bacterial infection, RhoGDI, Fig1G

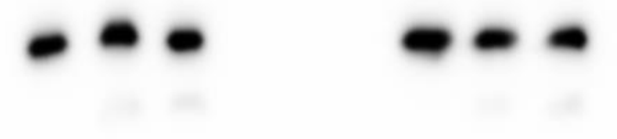

EV infection, RIG-I, Fig1H

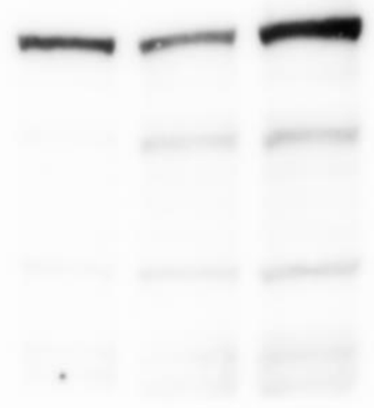

EV infection, IRAK1, Fig1H

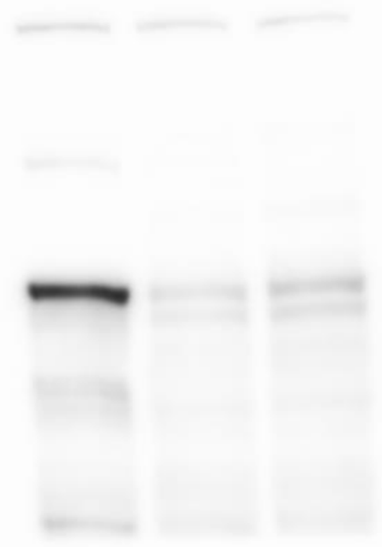

EV infection, p-IκBα, Fig4C

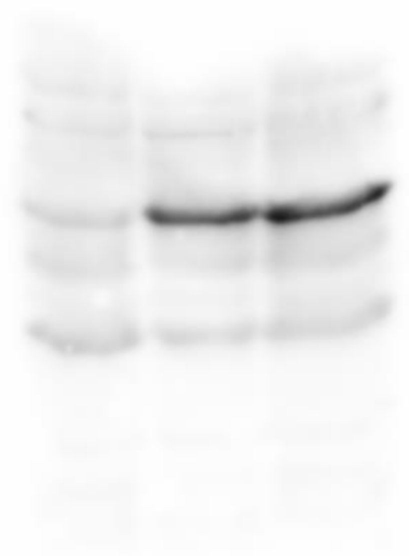

EV infection, p-RelA, Fig4C

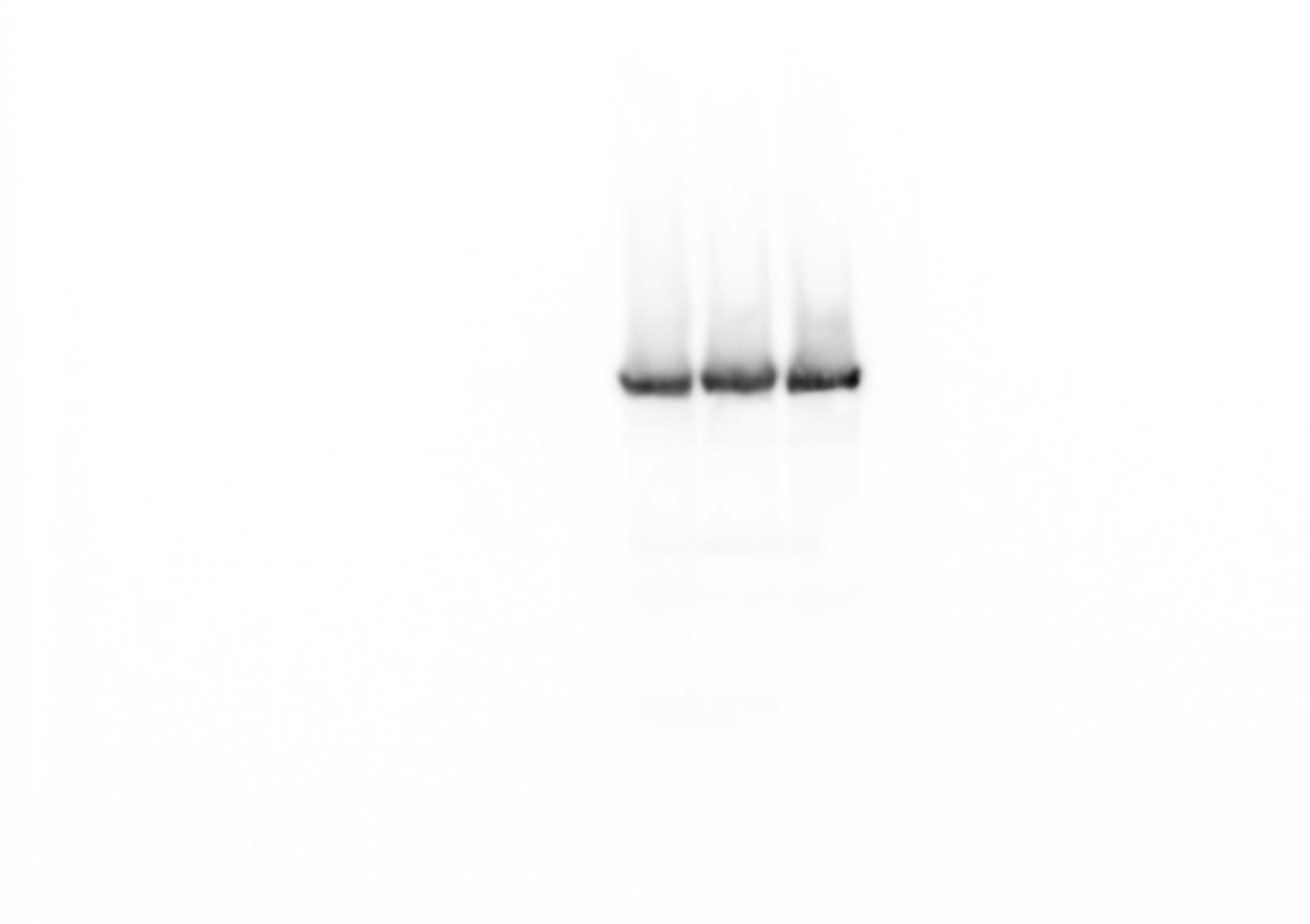

EV infection, cRel, Fig4C

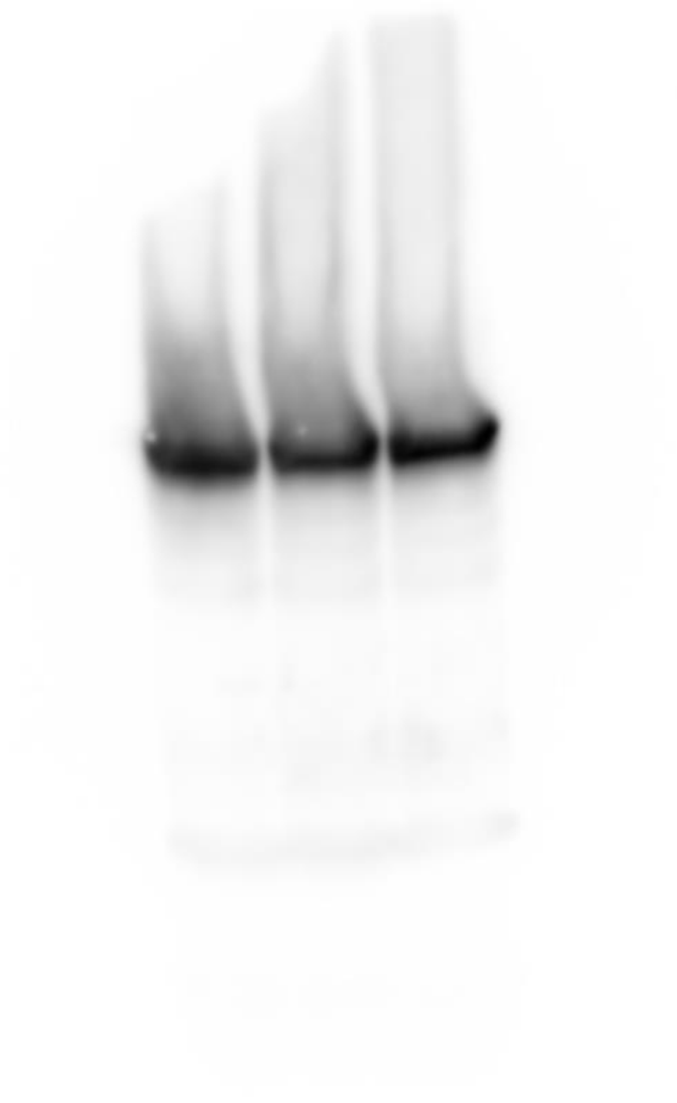

EV infection, Fig4C
p-TBK1(*) and p-IRF3(**)

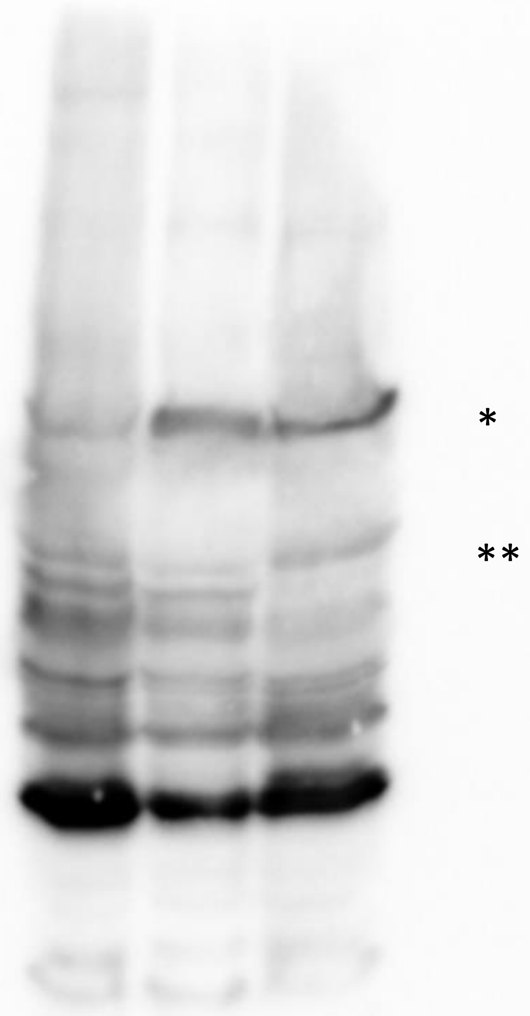

EV infection, p-IRF7(*), Fig4C

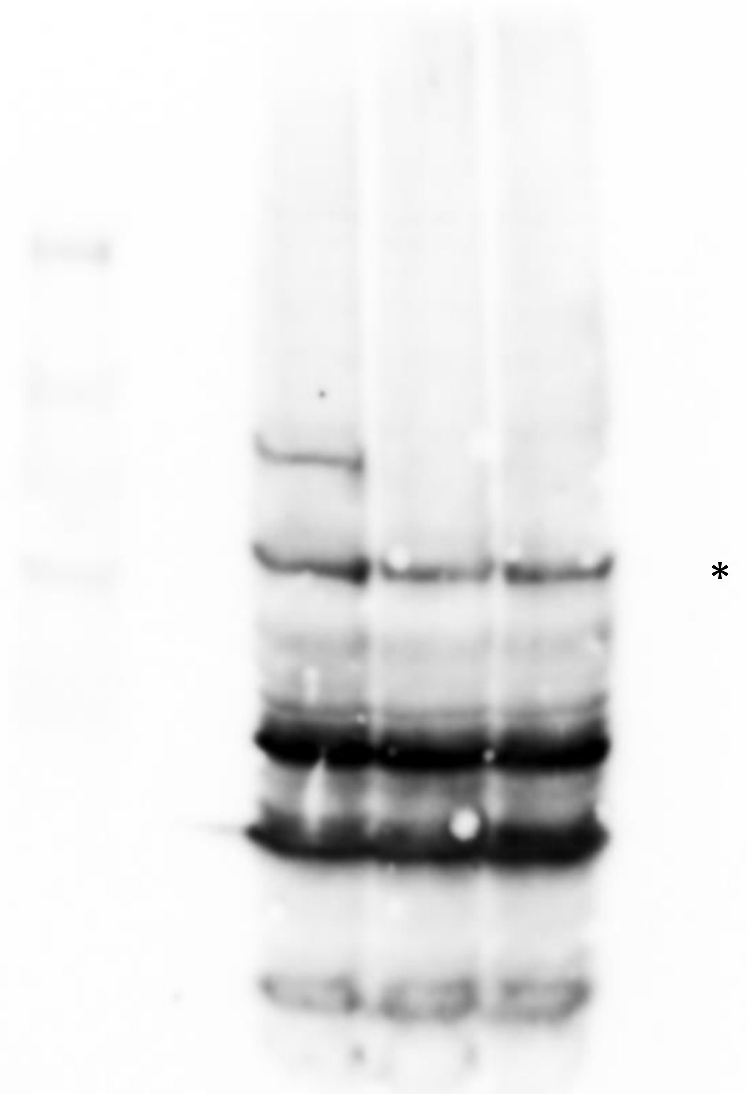

EV infection, RhoGDI_1, Fig1H

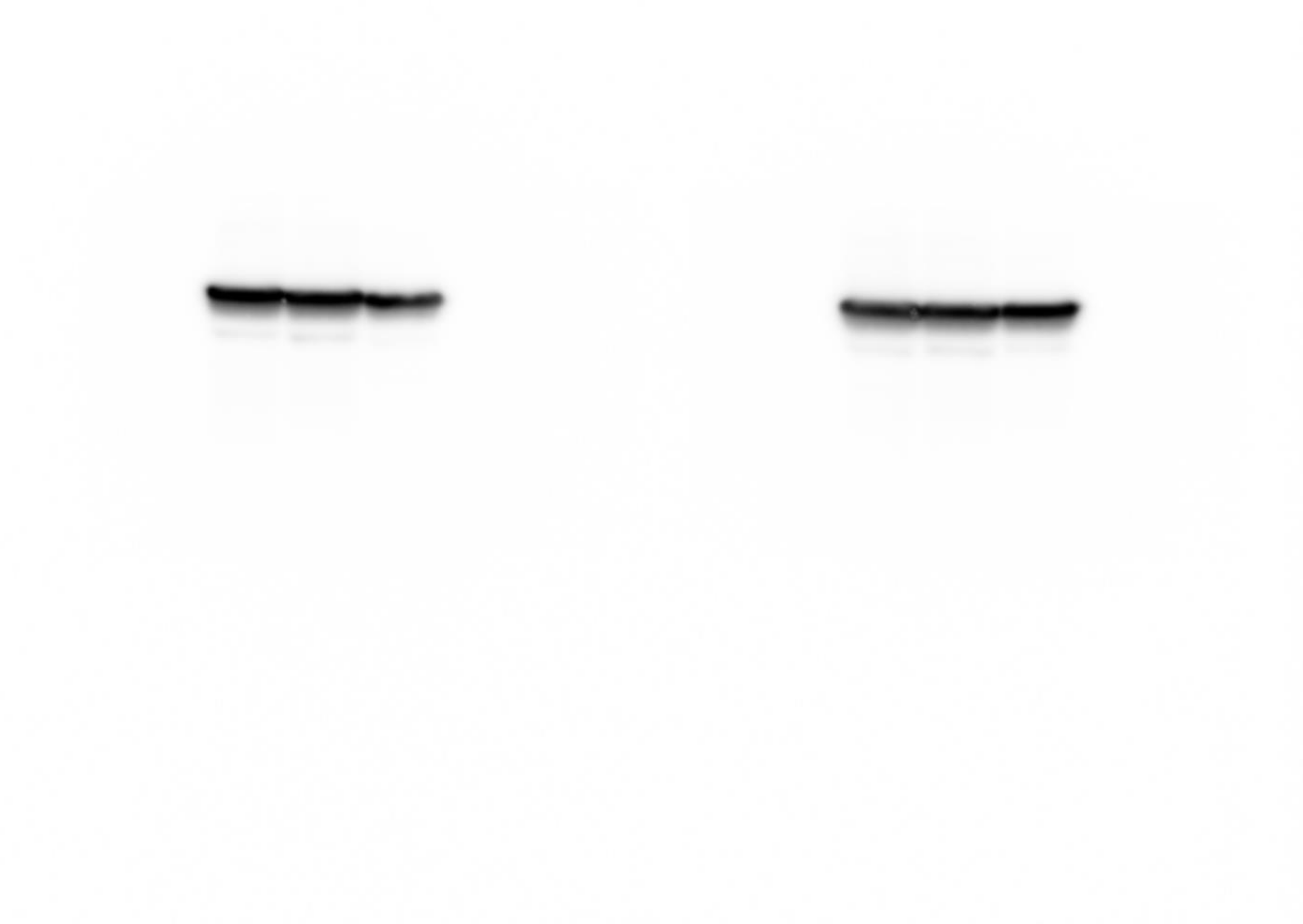

EV infection, RhoGDI_2, Fig4C

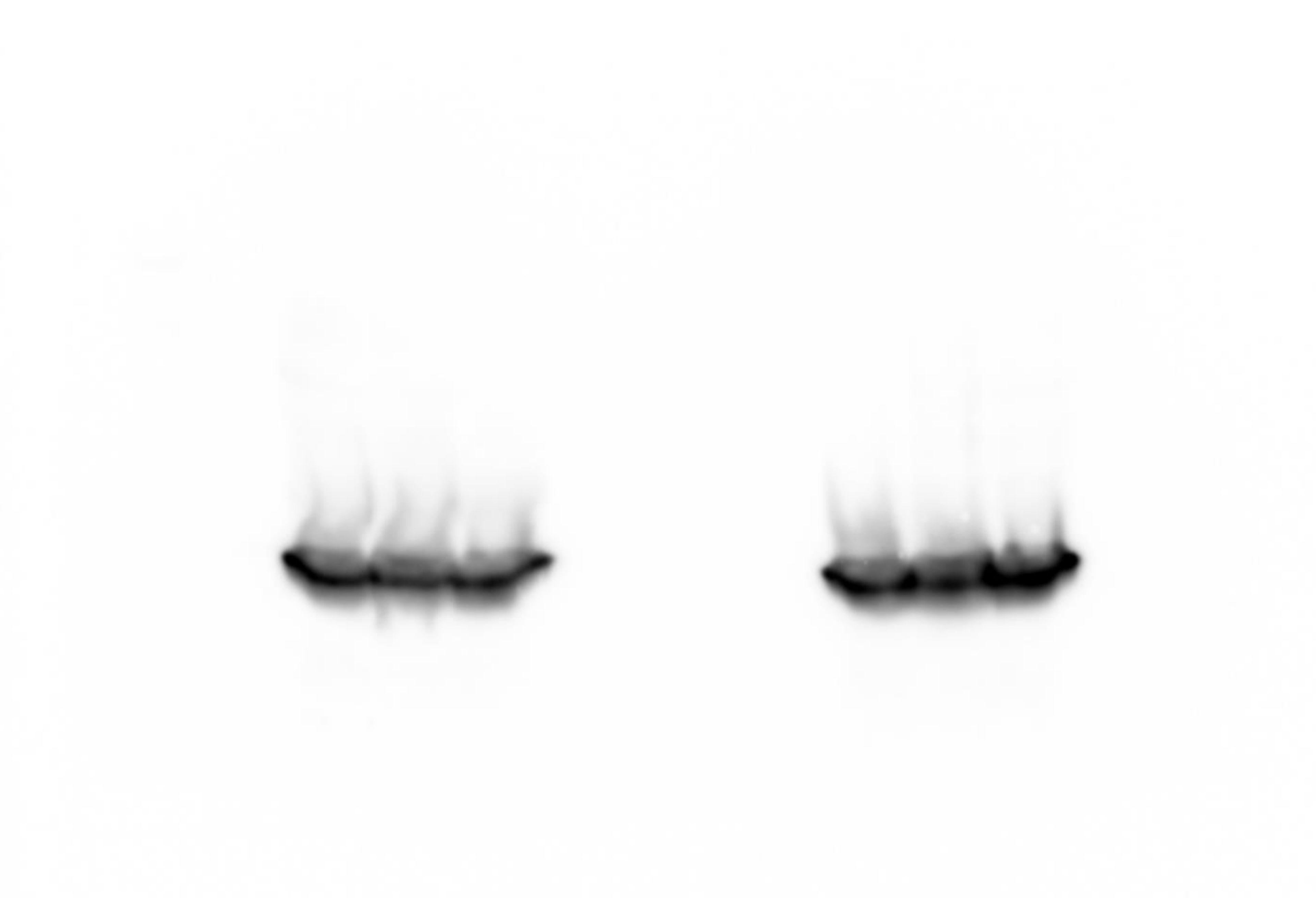

RNA transfection, RIG-I, Fig1I

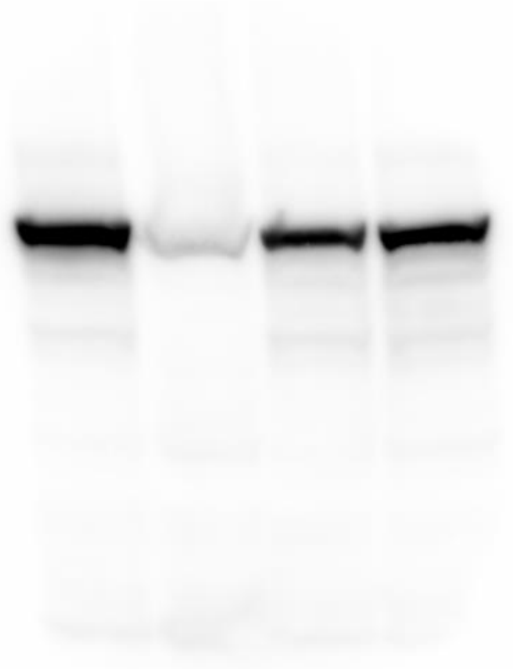

RNA transfection, IRAK1 (*), Fig1I

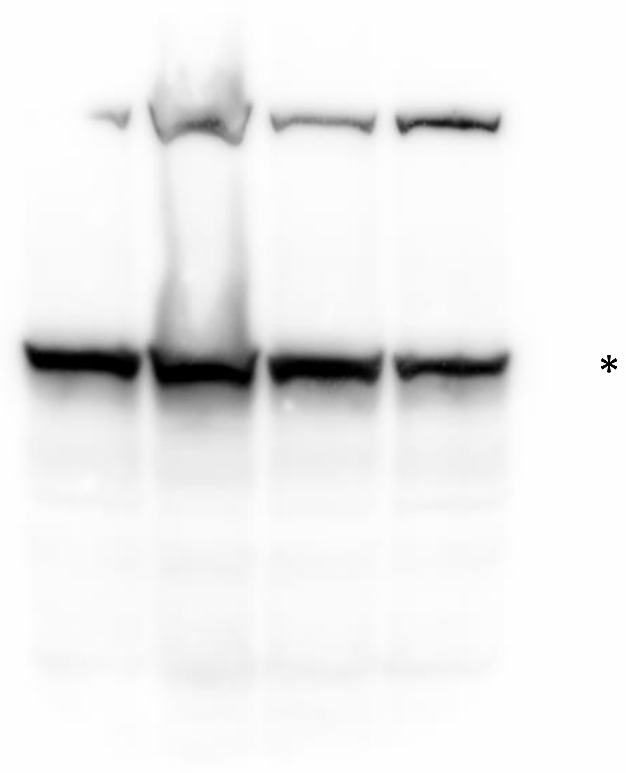

RNA transfection, cRel, Fig1I

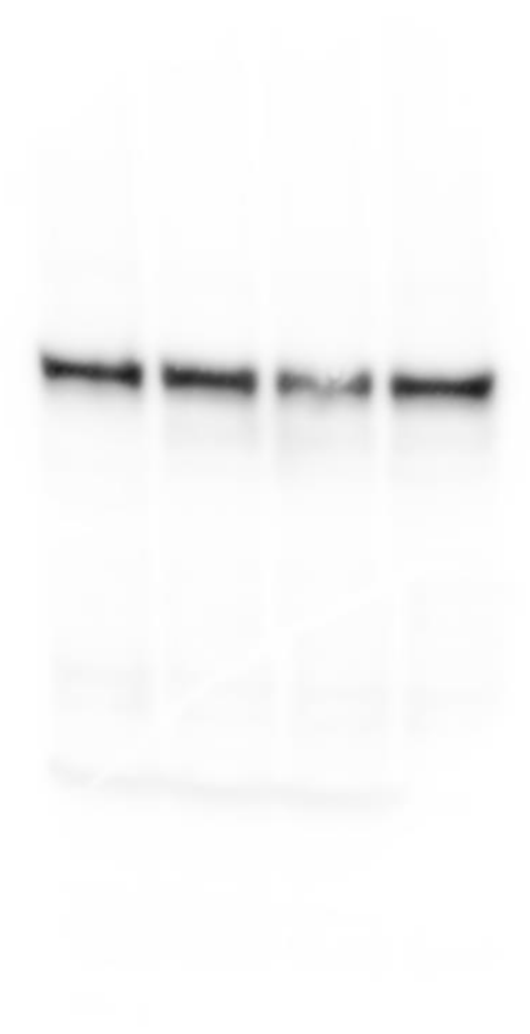

RNA transfection, RhoGDI, Fig1I

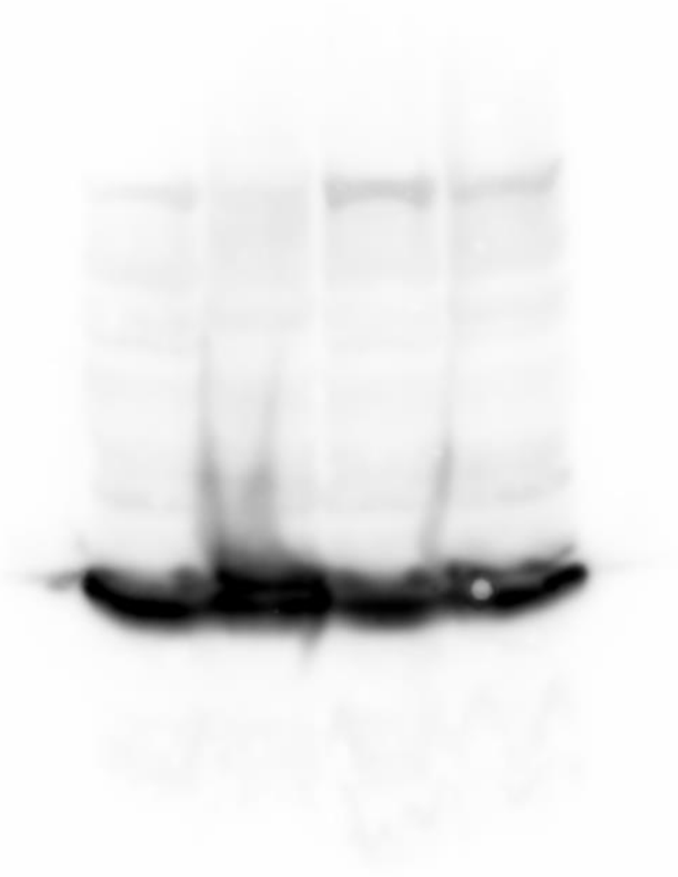

RsmY-RNA EMSA, Fig1J

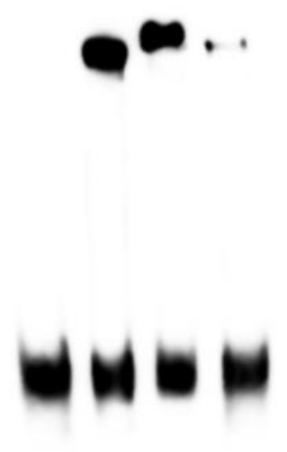

tRNA-Phe EMSA, Fig1J

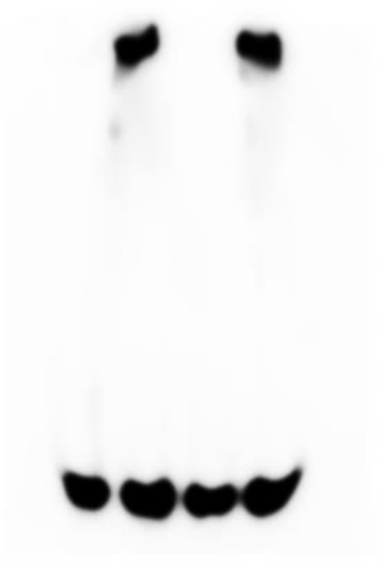

siRNA transfection, RIG-I

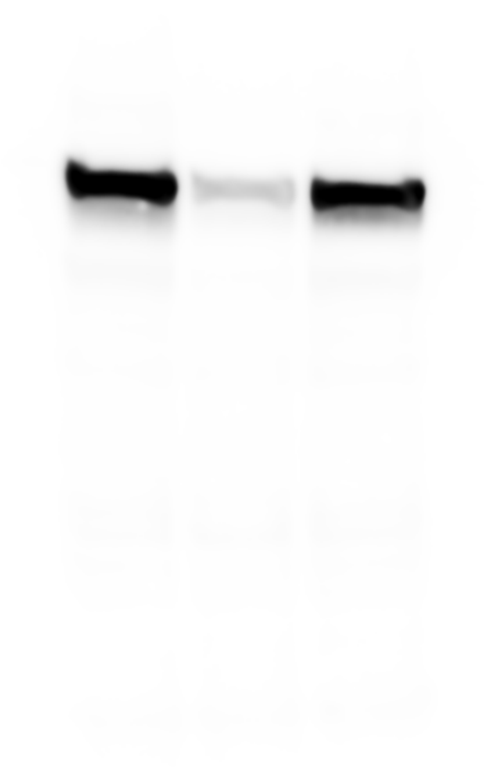

siRNA transfection, IRAK1

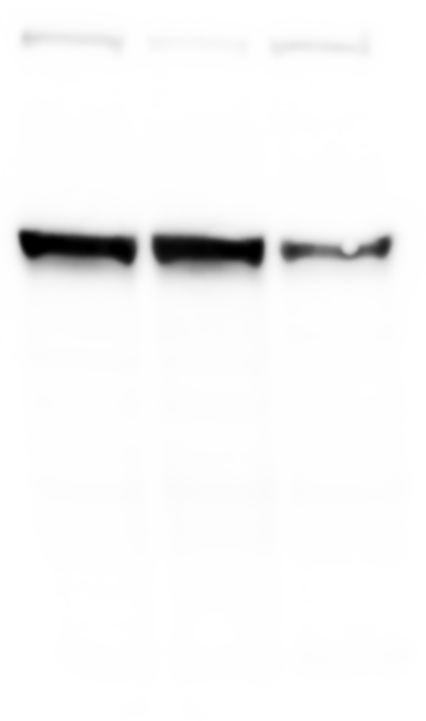

siRNA transfection, RhoGDI

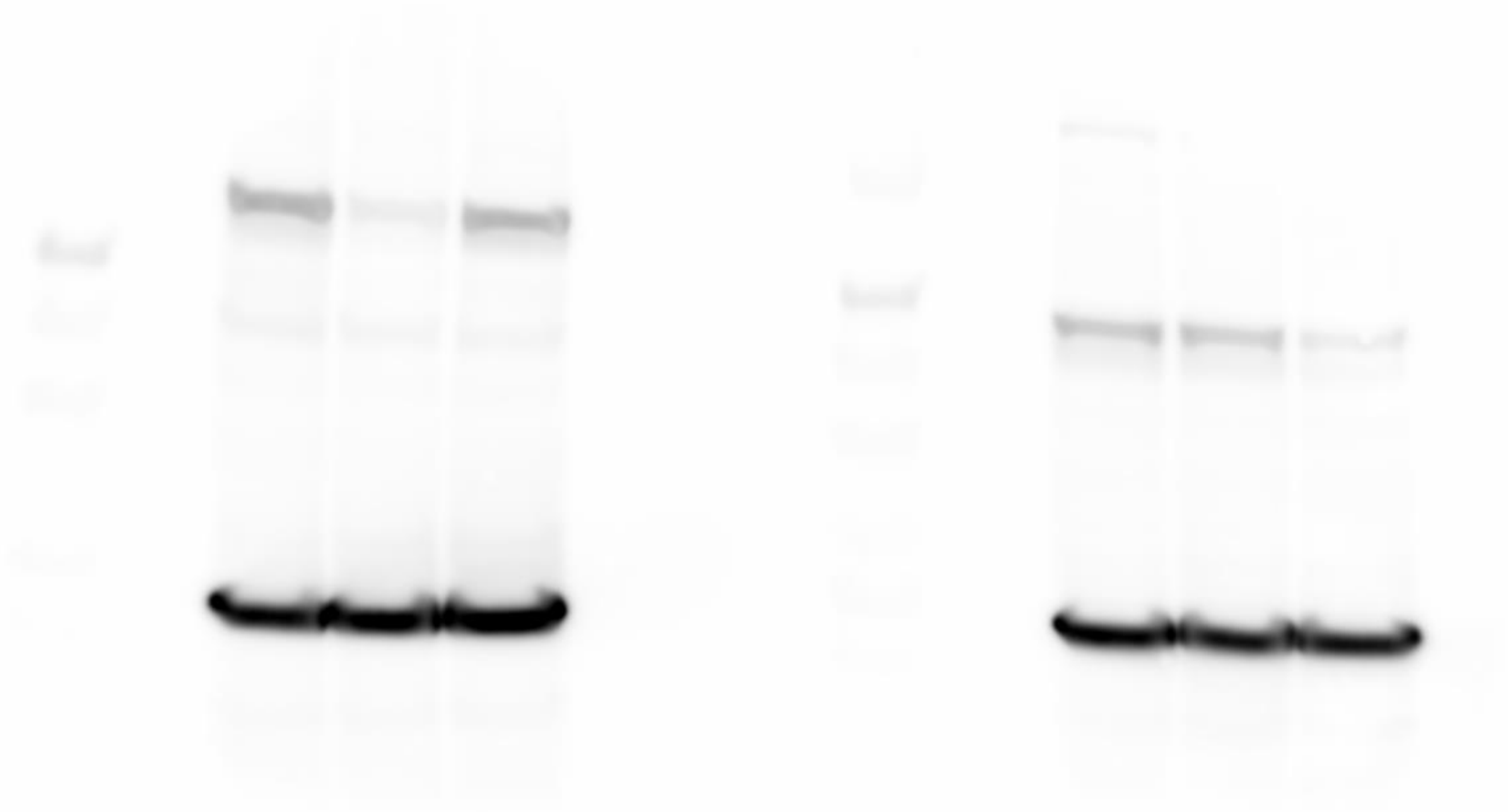

Supplement: Supplementary file 8 — Source Data [file 41467_2022_28454_MOESM8_ESM.zip › Source Data_Sahr/Sahr_RAW_images.pdf]
