# Supplementary material for: Translocated Legionella pneumophila small RNAs mimic eukaryotic microRNAs targeting the host immune response
Source: Nat Commun. 2022 Feb 9;13:762. doi: 10.1038/s41467-022-28454-x (PMC8828724; doi:10.1038/s41467-022-28454-x)

RNA transfection, RIG-I * and cRel ** (FigS4B)

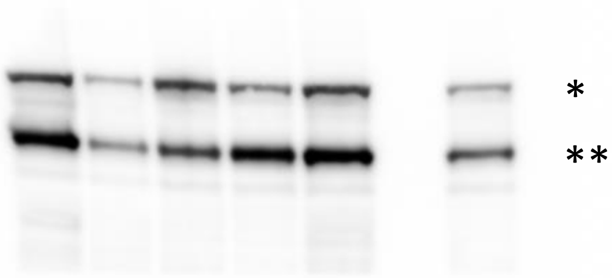

RNA transfection, IRAK1 (FigS4B)

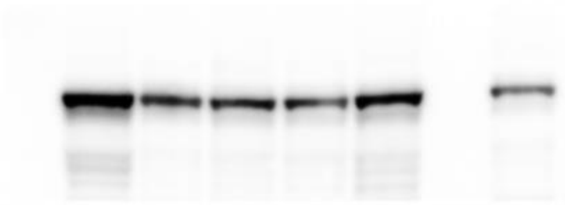

RNA transfection, RhoGDI (FigS4B)

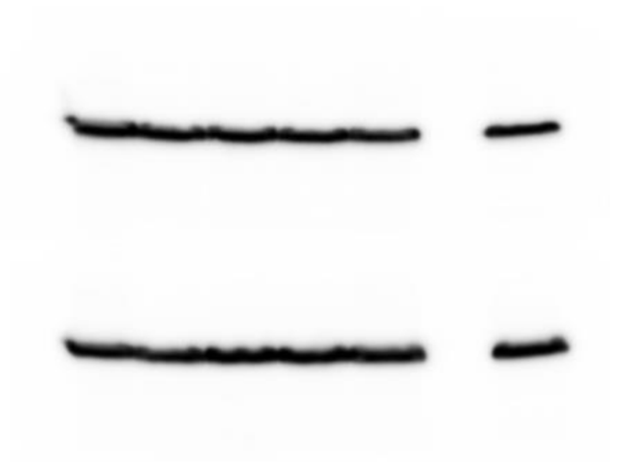

Bacterial infection, RIG-I_hMDM (FigS5)

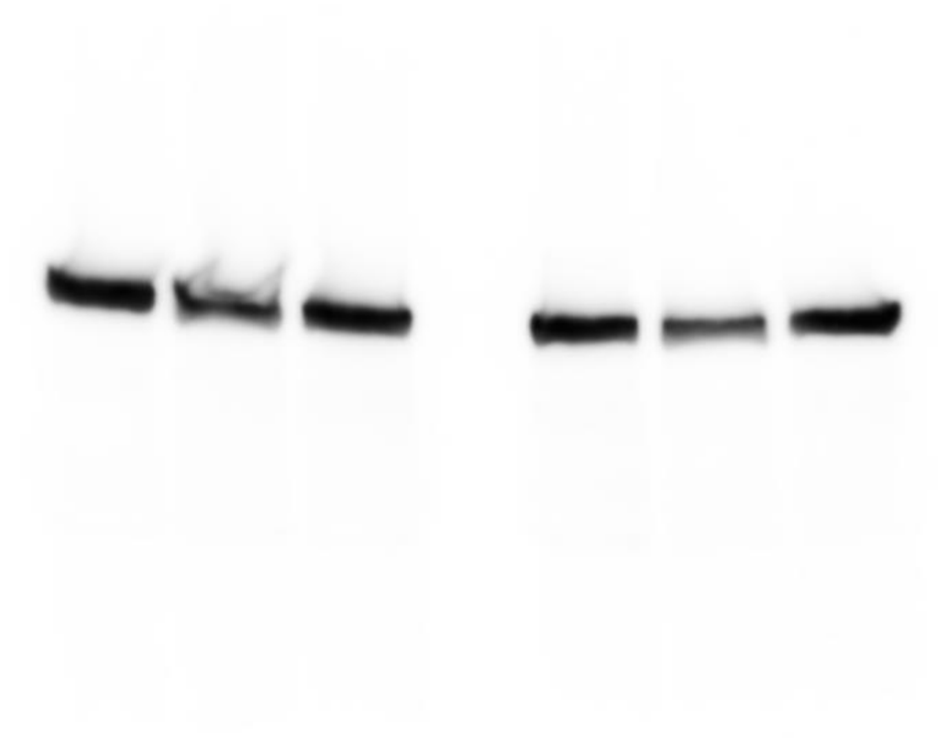

Bacterial infection, IRAK1_hMDM (FigS5)

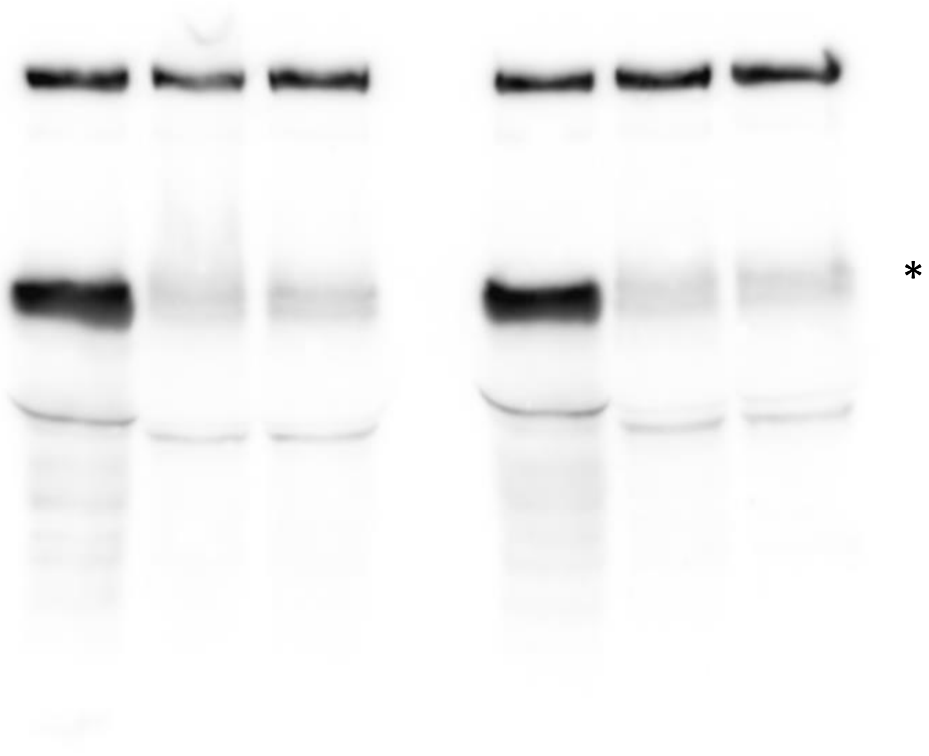

Bacterial infection, RhoGDI_hMDM (FigS5)

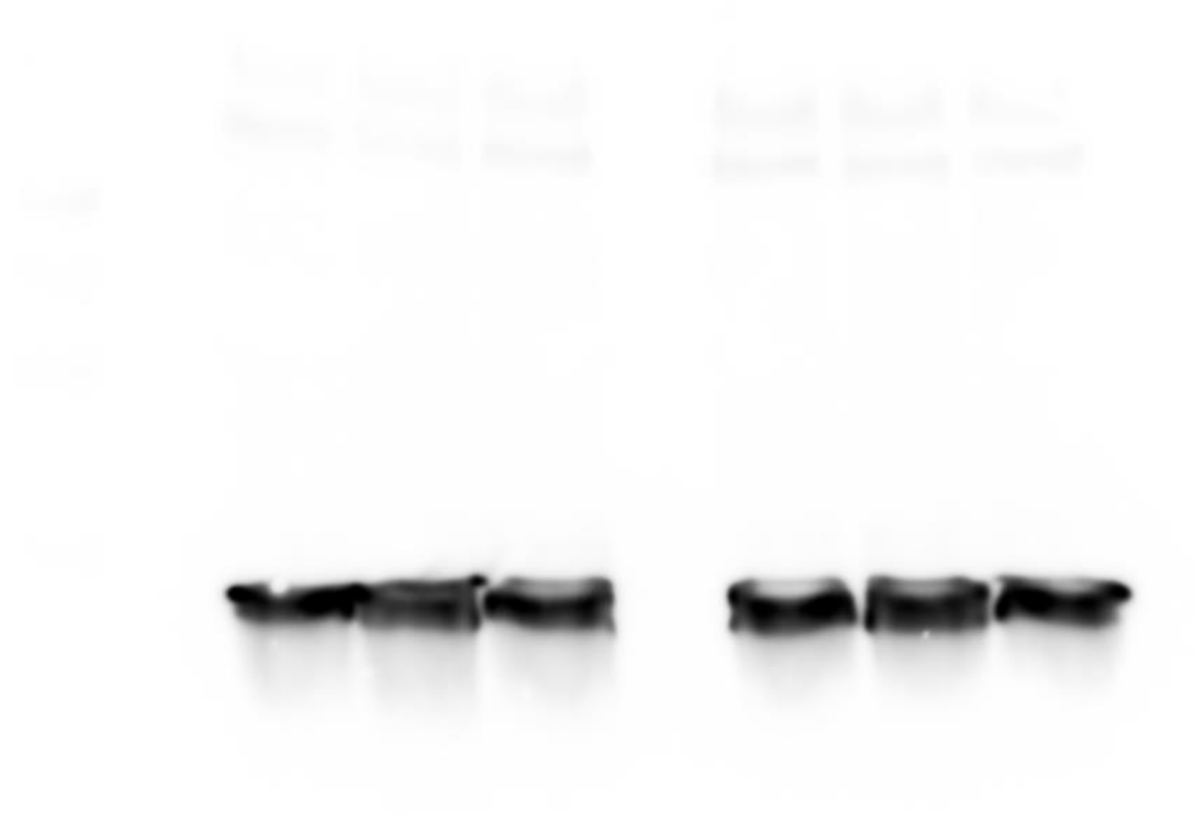

siRNA transfection, RIG-I

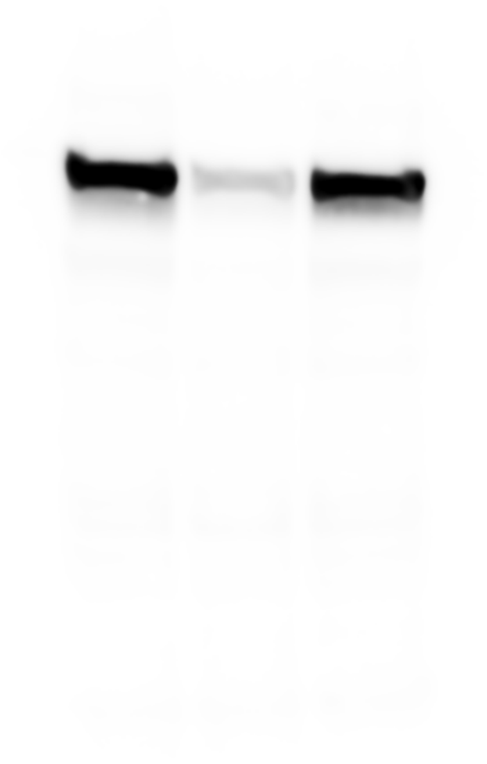

siRNA transfection, IRAK1

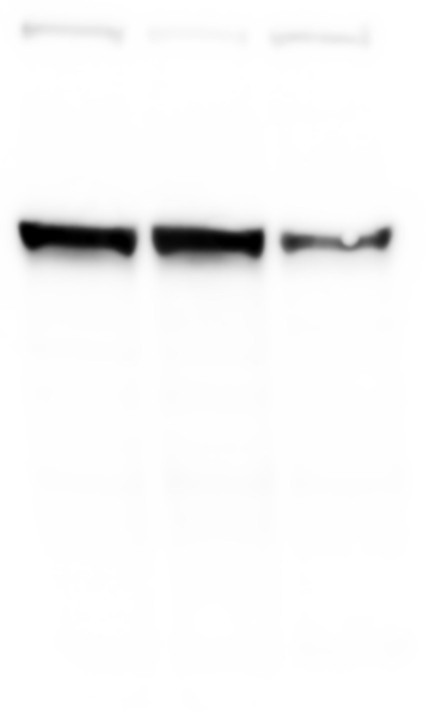

siRNA transfection, RhoGDI

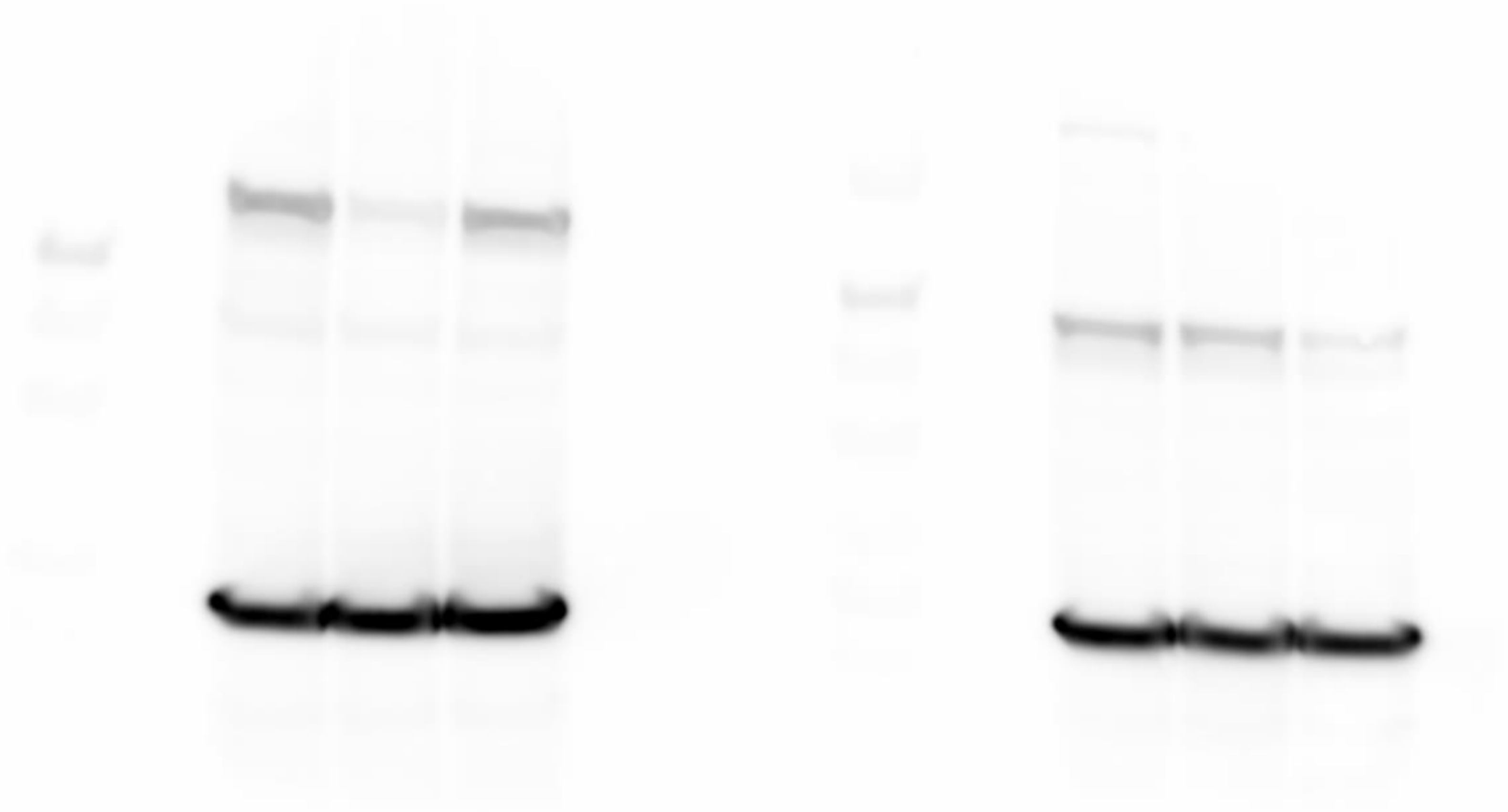

Supplement: Supplementary file 8 — Source Data [file 41467_2022_28454_MOESM8_ESM.zip › Source Data_Sahr/Sahr_Suppl RAW_images.pdf]
